# Lactate Dehydrogenase in Hepatocellular Carcinoma: Something Old, Something New

**DOI:** 10.1155/2016/7196280

**Published:** 2016-05-29

**Authors:** Luca Faloppi, Maristella Bianconi, Riccardo Memeo, Andrea Casadei Gardini, Riccardo Giampieri, Alessandro Bittoni, Kalliopi Andrikou, Michela Del Prete, Stefano Cascinu, Mario Scartozzi

**Affiliations:** ^1^Medical Oncology Unit, Università Politecnica delle Marche, AOU “Ospedali Riuniti Umberto I-G.M. Lancisi- G. Salesi”, 60121 Ancona, Italy; ^2^Medical Oncology Unit, Università degli Studi di Cagliari, AOU di Cagliari, 09042 Cagliari, Italy; ^3^Hôpitaux Universitaires de Strasbourg, Faculté de Médecine, IRCAD/EITS, Institut Hospitalo-Universitaire de Strasbourg IHU MixSurg, Université de Strasbourg, 67100 Strasbourg, France; ^4^IRCCS, Istituto Scientifico Romagnolo per lo Studio e la Cura dei Tumori, 47014 Meldola, Italy; ^5^Medical Oncology Unit, Università degli Studi di Modena e Reggio Emilia, 41121 Modena, Italy

## Abstract

Hepatocellular carcinoma (HCC) is the most common primary liver tumour (80–90%) and represents more than 5.7% of all cancers. Although in recent years the therapeutic options for these patients have increased, clinical results are yet unsatisfactory and the prognosis remains dismal. Clinical or molecular criteria allowing a more accurate selection of patients are in fact largely lacking. Lactic dehydrogenase (LDH) is a glycolytic key enzyme in the conversion of pyruvate to lactate under anaerobic conditions. In preclinical models, upregulation of LDH has been suggested to ensure both an efficient anaerobic/glycolytic metabolism and a reduced dependence on oxygen under hypoxic conditions in tumour cells. Data from several analyses on different tumour types seem to suggest that LDH levels may be a significant prognostic factor. The role of LDH in HCC has been investigated by different authors in heterogeneous populations of patients. It has been tested as a potential biomarker in retrospective, small, and nonfocused studies in patients undergoing surgery, transarterial chemoembolization (TACE), and systemic therapy. In the major part of these studies, high LDH serum levels seem to predict a poorer outcome. We have reviewed literature in this setting trying to resume basis for future studies validating the role of LDH in this disease.

## 1. Introduction

Hepatocellular carcinoma (HCC) is the most common primary liver tumour (80–90%) and represents more than 5.7% of all cancers. HCC incidence has risen to become the 5th commonest malignancy worldwide and the third leading cause of cancer-related death, after lung and stomach cancer. The estimated incidence of new cases is about 500,000–1000,000 per year, causing 600,000 deaths globally per year [1].

In the Western world, over 90% of HCC cases occur in cirrhotic liver, but globally, about 20% of HCC is not associated with any form of cirrhosis. In these cases, the etiology remains unknown. Main risk factors for the development of HCC can be classified into viral (chronic hepatitis B and hepatitis C), toxic (alcohol, aflatoxin), metabolic (diabetes, hemochromatosis, and nonalcoholic fatty liver disease), and immune-related (autoimmune hepatitis and primary biliary cirrhosis) [2] factors.

Chronic hepatitis and cirrhosis lead to a stepwise process that involves activation of oncogenes and inactivation of tumour suppressor genes through genetic and epigenetic alterations until HCC develops [2].

One particularly important characteristic of HCC in clinical practice is hypervascularization that modifies itself widely during the carcinogenesis process [3]. Several angiogenic proteins that influence neoangiogenesis and consequently tumour progression, high rate of metastasis, and bad prognosis of HCC have been identified [4–8].

However, the mechanism of neovascularization during HCC development is still not clear.

Liver tumours display a vasculature less dense than the normal liver. Some immature liver tumour vessels are excessively leaky and have abnormal blood flow. This results in hypovascular areas and severe hypoxia and/or necrosis. Hypoxia may promote growth of HCC and progression and resistance to therapies.

Hypoxia represents a clinical biological mechanism for treatment resistance in cancer cells via the formation of new blood vessels. Furthermore, a growing body of evidence indicates that hypoxia might actually promote cancer development.

Lactic dehydrogenase (LDH), which is a glycolytic enzyme, composed of four polypeptide chains, each one encoded by separate gene (M and H), exists in various types in human tissues and neoplasms. LDH is a key enzyme in the conversion of pyruvate to lactate under anaerobic conditions [9]. Five isoforms of LDH have been identified as a result of the five different combinations of polypeptide subunits [10].

LDH is typically released from necrotic cells. In several preclinical models investigating the role of tumour hypoxic microenvironment, a correlation between high tumour volume, high percentage of necrosis, high tumour LDH expression, and high serum LDH levels was, in fact, demonstrated. Upregulation of LDH has been suggested to ensure both an efficient anaerobic/glycolytic metabolism and a reduced dependence on oxygen under hypoxic conditions in tumour cells.

**Figure 1:**
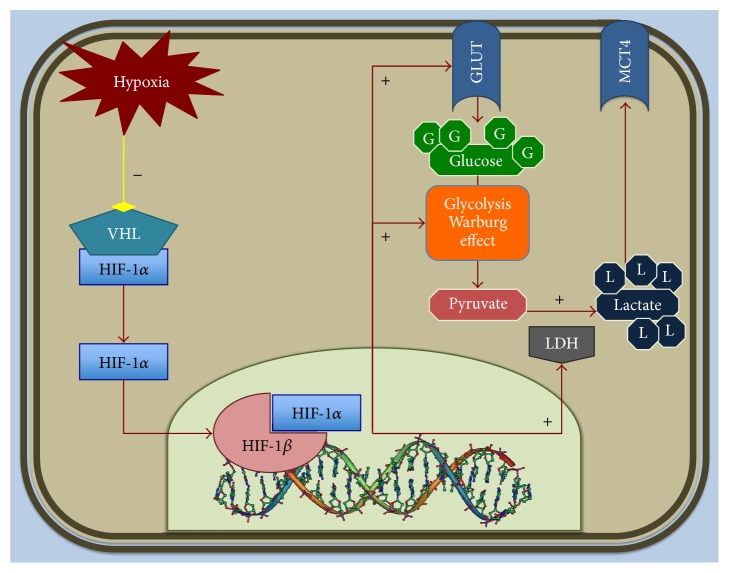
Effect of hypoxic microenvironment on metabolism of tumour cell. Under hypoxia conditions, VHL (Von Hippel Lindau suppressor) dissociates from subunit alpha of HIF-1. Thus, HIF-1*α* binds the beta subunit and promotes the nuclear transcription of several target genes (e.g., LDH) implicated in tumour angiogenesis, cell proliferation, and metabolism.

The biological link between hypoxia, LDH levels, and the tumour-driven angiogenesis pathway through the abnormal activation of the hypoxia inducible factor-1 (HIF-1) is well established (Figure 1). The biological activity of HIF-1 is determined by the expression and activity of the HIF-1*α* subunit [11]. HIF-1*α* is an essential factor that upregulates a series of genes involved in glycolytic energy metabolism, angiogenesis, erythropoiesis, and cell survival [12]. Hypoxia in the tumour microenvironment is sufficient to activate HIF-dependent expression of several downregulated genes [13]. These include those encoding for vascular endothelial growth factor, erythropoietin, and many enzymes involved in glucose, iron, and nucleotide metabolism [14].

Data from several analyses on different cancers seem to suggest that LDH levels may be a significant prognostic factor.

In colorectal cancer patients, LDH upregulation was in fact associated with an increased risk of nodal and distant metastases and high LDH serum levels have been shown to correlate with a decreased median overall survival [15–21].

A strong association between the expression of LDH and an aggressive phenotype has also been demonstrated in gastric cancer [22] and pancreatic cancer [23].

The role of LDH in HCC has been investigated by different authors in heterogeneous populations of patients. The aim of this work is to review the literature in this setting.

## 2. LDH in HCC Treated with Surgery

Although surgery remains a frequently used curative therapy for HCC, long term prognosis after liver resection remains unsatisfactory, due to high disease relapse incidence. Tumour recurrence may originate from either intrahepatic metastasis of primary HCC or de novo carcinogenesis from the remnant cirrhotic liver [24].

Early HCC recurrence after hepatectomy is associated with worse clinical outcome. Identification of patients at high risk for early disease relapse may help improving the prognosis of this population by surveillance and well-timed treatment of recurrent disease. Although several studies suggested factors related to tumour [24, 25] and to treatment [26, 27] as risk factors for early recurrence of HCC, the use of these parameters is technically problematic and cannot be easily used to predict recurrence risk in daily practice.

LDH serum levels as prognostic factor were investigated in different studies in early HCC patients treated with hepatic resection.

In a retrospective study on 200 patients treated with curative hepatic resection, Wang et al. [28] evaluated several serum and clinical factors collected at baseline before treatment. Patients were divided according to the median recurrence free survival (RFS) in early recurrence group (ER) and nonearly recurrence group (non-ER). At multivariate analysis, five independent adverse prognostic factors for early recurrence were identified: LDH (HR = 1.711, 95% CI = 1.170–2.502, and *p* = 0.006), aspartate aminotransferase (AST)/alanine aminotransferase (ALT) ratio (HR = 1.769, 95% CI = 1.180–2.540, and *p* = 0.006), alpha-fetoprotein (AFP) (HR = 2.079, 95% CI = 1.221–3.542, and *p* = 0.007), resection margin (HR = 2.354, 95% CI = 1.490–3.719, and *p* < 0.001), and TNM stage (HR = 2.164, 95% CI = 1.463–3.201, and *p* < 0.001).

The role of serum presurgery LDH levels was confirmed in another larger study (323 patients) by Hu et al. [29]. Patients were categorized as high LDH (>240 U/L) and low LDH group (≤240 U/L). Significant differences in tumour size, capsulation, tumour number, vascular invasion, and TNM stage were observed between these two groups (*p* < 0.05). The 1-, 3-, 5-year disease-free survival (DFS) (24.3%, 13.5%, and 12.2% versus 51.2%, 36.3%, and 32.1%; *p* < 0.001) and overall survival (45.9%, 28.4%, and 24.3% versus 78.3%, 51.8%, and 43.7%; *p* < 0.001) of HCC patients in the LDH > 240 U/L group were poorer than those in the LDH ≤ 240 U/L group. Multivariate analysis demonstrated that LDH > 240 U/L served as an independent prognostic indicator of worse disease-free survival (HR = 1.711, 95% CI = 1.275–2.297, and *p* < 0.001) and overall survival (HR = 1.568, 95% CI = 1.144–2.149, and *p* = 0.005). Stratification analysis showed that LDH exhibited a greater predictive value for DFS and OS in HCC patients with AFP < 200 ng/mL.

Although these studies have included heterogeneous populations of patients, LDH serum levels emerge as a useful prognostic marker after liver resection.

## 3. HCC Treated with Transarterial Chemoembolization (TACE)

Surgery is the only potentially curative treatment for HCC; unfortunately, most patients in Western countries present with an intermediate or advanced HCC at diagnosis with the consequent impossibility of undergoing curative treatments. These patients are therefore candidates to palliative therapies such as arterial embolization, chemoembolization (TACE), and systemic treatment [30]. TACE represents a crucial treatment option for HCC; however, comparing clinical findings, results are often hampered by the considerable variability in patients' selection criteria and modalities of execution of therapy [31–34]. However, global results for TACE are still insufficient, with only a small proportion of patients benefiting from these procedures. The molecular mechanism that accounts for treatment failure is not clear [35, 36]. It is possible that some adaptive responses to hypoxia may represent a key factor for resistance. Starting from these assumptions, the role of LDH has also been evaluated in this category of patients.

Kohles et al. showed a possible prognostic role for pretreatment LDH serum levels in HCC patients undergoing TACE [37]. Levels of liver-specific, tumour-related, and cell death biomarkers were analyzed and correlated with overall patient survival on 50 prospectively and consecutively HCC patients undergoing TACE. Serum levels were collected before and 24 hours after TACE application. At univariate analysis, high levels of cytokeratin 19-fragments (CYFRA 21-1), AFP, and low cholinesterase (CHE) levels measured before and 24 hours after TACE were correlated with unfavorable outcome. Further high pretherapeutic LDH, AST, and bilirubin levels as well as high 24-hour C-reactive protein values were associated with poor survival. At multivariate analysis of clinical and only pretherapeutic biomarkers, AFP, CHE, and LDH have been shown to be independent prognostic parameters. When additionally 24-hour values were included, CHE (24 h) and AFP (24 h) were the strongest independent prognostic biomarkers with a slightly higher prognostic power.

In this setting, the role of LDH was evaluated also by our group in a retrospective study [38]. We analyzed a population of 114 HCC consecutive patients, treated with TACE from 2002 to 2010, at our institution. Patients were classified according to ECOG PS (Eastern Cooperative Oncology Group performance status) and were staged using different staging systems: Child-Pugh, BCLC, Okuda, MELD (Model for End-Stage Liver Disease), and MELD-Na (Model for End-Stage Liver Disease, Sodium). We recorded LDH serum levels before (within 1 month prior to treatment) and after (within one month after) treatment.

Patients were divided into two groups, according to LDH serum concentration registered before TACE. First group included patients with pretreatment LDH ≤ upper normal limit of 450 U/L (group A), whereas the other group included patients with pretreatment LDH > 450 U/L (group B). Patients were, also, classified according to any variation in LDH serum levels before and after treatment (increased versus decreased).

In patients with LDH values below 450 U/L, median time to progression (TTP) was 16.3 months, whereas it was 10.1 months in patients above the cut-off (*p* = 0.0085). Accordingly, median overall survival (OS) was 22.4 months and 11.7 months in groups A and B, respectively (*p* = 0.0049). In patients with decreased LDH values after treatment, median TTP was 12.4 months, and median OS was 22.1 months, whereas TTP was 9.1 months and OS was 9.5 in patients with increased LDH levels (TTP: *p* = 0.0087; OS: *p* = 0.0001). No statistically significant differences were found between the groups of patients for all clinical characteristics analyzed (gender, median age, performance status ECOG, staging systems, and type of TACE performed).

From these experiences, although it is based on small series, it is clear that patients stratification may represent a crucial factor for the choice of the appropriate treatment strategy for the appropriate patient. LDH serum levels have established the potential to predict clinical outcome and consequently to lead to better patients selection in this clinical scenario as well.

## 4. LDH in HCC Treated with Systemic Therapies

In the last few years, the introduction of sorafenib, an oral multityrosine kinase inhibitor (TKI) for the treatment of advanced HCC patients, changed the clinical landscape for these tumours and now represents the standard of care [39–42]. However, a large proportion of patients still do not seem to benefit from such a treatment approach and are therefore exposed to unnecessary toxicity [39–42].

Clinical or molecular criteria allowing a more accurate selection of resistant/responder tumours are in fact largely lacking, although they would be obviously crucial for an optimal management of these patients in the clinical practice [43].

In preclinical studies, high levels of LDH were reported to predict resistance to several tyrosine kinase inhibitors (TKI), including sorafenib [44].

It has been demonstrated that the inhibition of LDH production with oxamic acid in cancer cell lines potentiated the antiproliferative activity of tyrosine kinase inhibitors, such as sorafenib. The effect of high LDH levels on TKI low activity may be explained by a competition between ATP and TKIs inhibition at the ATP enzymatic site on the protein kinases target of their activity. LDH catalyzed the final step in the glycolytic pathway, the conversion of pyruvate and NADH to lactate and NAD+, determining the maintenance of glycolytic flow, and, consequently, the production of ATP. In cancer cells, in hypoxic conditions, in which anaerobic glycolysis is the main metabolic pathway to meet the energy request, the inhibition of LDH could interfere with this process, causing the depletion of ATP and therefore a lower competition against TKIs inhibitors.

On this basis, we have conducted a retrospective study to evaluate the clinical role of LDH in 78 Child-Pugh A advanced HCC patients treated with sorafenib [45].

We have recorded LDH serum levels before (within 1 month prior to the start of sorafenib treatment) and after (within one month after the end of sorafenib treatment) treatment. The cut-off point with the highest sensitivity and specificity for estimating pretreatment LDH serum levels as a function of treatment clinical activity was set after ROC curve analysis at ≤407 U/L for both PFS and OS.

At univariate analysis, in patients with LDH values below the cut-off, median PFS was 6.7 months, whereas it was 1.9 months in patients above the cut-off (HR = 2.79, 95% IC = 1.27–6.15, and *p* = 0.0002). Similarly, median OS was 13.2 months and 4.9 months in the two groups (HR = 2.74, 95% IC = 1.22–6.16, and *p* = 0.0006). In patients with decreased LDH values after treatment, median PFS was 6.8 months, and median OS was 21.0 months, whereas PFS was 2.9 months and OS was 8.6 months in patients with increased LDH levels (PFS: HR = 0.48, 95% IC = 0.27–0.84, and *p* = 0.0087; OS: HR = 0.42, 95% IC = 0.23–0.65, and *p* = 0.0035). At multivariate analysis of LDH serum levels before treatment, the variation after treatment and BCLC stage emerged as independent prognostic factors predicting outcome in terms of PFS (*p* = 0.0197, HR = 0.71; *p* = 0.0201, HR = 0.19; and *p* = 0.0016, HR = 0.35, resp.) and OS (*p* = 0.0011, HR = 0.69; *p* = 0.0039, HR = 0.24; and *p* = 0.0051, HR = 0.39, resp.).

Another Italian study tried to verify the role of LDH in this setting. Analysis on a population of 97 HCC patients treated with sorafenib, part of the ITA.LI.CA (Italian Liver Cancer) database, seems to contest our findings [46].

Patients with LDH values above (*n* = 45) and below (*n* = 52) the cut-off (297 U/L) showed equal OS (12.0 months) and TTP (4.0 months) values. Data on LDH levels during sorafenib treatment were reported for 10 patients. LDH values decreased in 3 patients (mean difference = −219 U/L) who also reported a prolonged OS and TTP versus those with unmodified/increased LDH (OS: NE (not evaluated) versus 8.0 months, *p* = 0.0083; TTP: 19.0 versus 3.0 months, *p* = 0.008).

In this study, the clinical benefits of sorafenib do not seem to be influenced by baseline LDH levels; however, a decreased LDH concentration during sorafenib might be associated with improved clinical outcomes.

## 5. Other Studies on Heterogeneously Treated Populations

Other studies had evaluated the role of LDH in HCC patients not selected for the treatment received.

In a retrospective analysis on 273 HCC patients treated with different therapies (resective surgery, transarterial chemoembolization, sorafenib, and radiotherapy) by Yang et al., patients were divided into two groups: death and alive at the time of the study [47]. Among the liver function tests, levels of alanine aminotransferase (AST), gamma-glutamyl transferase (GGT), alkaline phosphatase (ALP), and LDH were statistically higher in patients with death outcome (all *p* < 0.05).

At global multivariate survival analysis, of all clinical and serological factors evaluated, ALT, GGT, LDH, carcinoembryonic antigen (CEA) levels, and Barcelona Clinic Liver Cancer (BCLC) stage were significantly associated with HCC overall survival (HR = 1.01, 95% CI = 1.002–1.017, and *p* = 0.01; HR = 1.003, 95% CI = 1.001–1.004, and *p* < 0.001; HR = 1.003, 95% CI = 1.002–1.005, and *p* < 0.001; HR = 1.015, 95% CI = 1.005–1.025, and *p* = 0.003; and HR = 2.428, 95% CI = 1.458–4.044, and *p* = 0.001, resp.).

Another larger retrospective analysis, evaluating clinical e biological factors, was conducted on a South Korean population of 743 HCC patients [48]. On multivariate analysis, LDH <450 IU/L and other factors like age >50 years, CLIP score <3, ALP <120 U/L, CRP <0.8 mg/dL, tumour size <6 cm, no distant metastasis, and curative treatment modality were predictors for 1-year survival.

## 6. Conclusions

In the investigation of predictive and prognostic factors for relatively rare clinical conditions such as HCC, finding data based on large and homogeneous series is particularly challenging. Furthermore, HCC is a complex disease; in most cases, two pathologic conditions, the tumour and the underlying liver disease, coexist in the same patient and have a predominant influence on clinical outcome.

All studies reported are retrospective and, in most of them, patients enrolled were in different tumour and liver function stages. Patients were stratified according to different characteristics in each study and data were collected in multicentre series in a large amount of time.

Moreover, the major parts of the studies reviewed are not designed to validate the LDH role. But LDH is studied among other serum or clinical factors, trying to find out some potential prognostic markers.

Among all studies taken into account, statistical methods are not reproducible. For example, the method used to choose the cut-off to divide population into high or low LDH is different in each study. Thus, it is possible that the potential role of LDH could be underestimated.

Despite all criticism cited, LDH seems to show a potential clinical role. It should be important to try to validate the role of LDH in clinical practice. In fact, LDH could be considered an ideal biomarker, easily obtained in every laboratory, reproducible, and low costing.

Prospective, upfront stratified, LDH-based trials are needed to confirm the power of this marker as predictive and prognostic factor in HCC patients.

After these confirmations, we believe that LDH should be considered as a relevant biological variable to be included in the baseline setup of HCC patients, with the aim to better stratify patients included in clinical trials and to better define the most appropriate therapeutic strategy.
